# Inert Gas Deactivates Protein Activity by Aggregation

**DOI:** 10.1038/s41598-017-10678-3

**Published:** 2017-08-31

**Authors:** Lijuan Zhang, Yuebin Zhang, Jie Cheng, Lei Wang, Xingya Wang, Meng Zhang, Yi Gao, Jun Hu, Xuehua Zhang, Junhong Lü, Guohui Li, Renzhong Tai, Haiping Fang

**Affiliations:** 1 Key Laboratory of Interfacial Physics and Technology, Chinese Academy of Sciences, Shanghai, 201800 China; 2 Shanghai Synchrotron Radiation Facility, Shanghai Institute of Applied Physics, Chinese Academy of Sciences, Shanghai, 201204 China; 3 State Key Laboratory of Molecular Reaction Dynamics, Dalian Institute of Chemical physics, Chinese Academy of Sciences, Dalian, 116023 China; 4 Division of Interfacial Water, Shanghai Institute of Applied Physics, Chinese Academy of Sciences, Shanghai, 201800 China; 5 Division of Physical Biology, Shanghai Institute of Applied Physics, Chinese Academy of Sciences, Shanghai, 201800 China; 6 University of the Chinese Academy of Sciences, Beijing, 100049 China; 7 Institute of Mathematics and Physics, Central South University of Forestry and Technology, Changsha, 410004 China; 8 Soft Matter & Interfaces Group, School of Engineering, RMIT University, Melbourne, VIC 3001 Australia

## Abstract

Biologically inert gases play important roles in the biological functionality of proteins. However, researchers lack a full understanding of the effects of these gases since they are very chemically stable only weakly absorbed by biological tissues. By combining X-ray fluorescence, particle sizing and molecular dynamics (MD) simulations, this work shows that the aggregation of these inert gases near the hydrophobic active cavity of pepsin should lead to protein deactivation. Micro X-ray fluorescence spectra show that a pepsin solution can contain a high concentration of Xe or Kr after gassing, and that the gas concentrations decrease quickly with degassing time. Biological activity experiments indicate a reversible deactivation of the protein during this gassing and degassing. Meanwhile, the nanoparticle size measurements reveal a higher number of “nanoparticles” in gas-containing pepsin solution, also supporting the possible interaction between inert gases and the protein. Further, MD simulations indicate that gas molecules can aggregate into a tiny bubble shape near the hydrophobic active cavity of pepsin, suggesting a mechanism for reducing their biological function.

## Introduction

Despite their biologically inert nature, inert gases still play important roles in a diverse range of biological processes including analgesia, anaesthesia, neuroprotection, tissue protection, antiapoptoticity, anxiolyticity, cytoprotection, ischemic-perfusion injury prevention, anticonvulsion, memory, drug addiction, and more^1–5^.

To date, Xe and Kr have been reported to bind preferentially to hydrophobic regions in proteins and membranes, acting as possible probes for investigating the structure–function relationship of the protein^6–9^. They have also proven useful for defining pathways for the diffusion of the ligand/substrate to an active site^10, 11^. It has been reported that even weak nonspecific interactions between Xe and protein surfaces have a measurable effect on the chemical shift of Xe, and that this effect changes upon protein denaturation^12, 13^. Franks *et al*. reported that Xe inhibits NMDA receptors based on the measurement of NMDA-activated currents in cultured hippocampal neurons^14^. More recently, Turin *et al*. claimed that Xe causes an electron spin change during general anaesthetics in drosophila, as do some non-inert gas molecules of sulfur hexafluoride, nitrous oxide, and chloroform^15^. N_2_ at a high concentration in body tissues could cause narcosis effects^16–18^.

Although the intriguing biological effects of inert gases have been known for centuries^2, 14, 15^, researchers are challenged to understand the mechanisms behind these effects, as inert gases are very chemically stable and are not easily absorbed on biological molecules due to a weak interaction energy. The inert gas binds directly to proteins via a London dispersion of typically ~1–2 kcal/mol, and it is easily removed from the surface by thermal fluctuations^19, 20^. Although the Xe-protein interaction might be enhanced by the charge-induced dipole due to its diffusive electron cloud, the biological effects induced by other biologically inert gases such as N_2_ and Kr remain difficult to understand^21, 22^.

Herein, pepsin and biologically inert gases, Kr, Xe and N_2_ are used as model systems to both experimentally and numerically investigate the gas aggregation in a pepsin solution and the impact of inert gases on the biological activity of a protein. Pepsin is the principal proteolytic enzyme in the stomach, and it has been widely used to investigate the toxicity mechanism of many drugs and chemical components^23^. Pertinent to this study, pepsin has a large apolar cavity, which is presumed to be the main binding site for gas molecules. Initially, micro X-ray fluorescence absorption spectra is employed to measure the absorption intensity of Kr and Xe in a pepsin solution after gassing and degassing. Enzymatic activity experiments are subsequently explored to study the ability of Kr, Xe, and N_2_ to inhibit pepsin activity. Nanosight particle tracking experiments for the system gassed by Xe provides the distribution of sizes and numbers of Xe-associated “nanoparticles”. The MD simulation further indicates the binding sites and states of those inert gases on the pepsin molecule, which differ by gas type. Both modelling and experimental results indicate that the aggregation of inert gases (Kr, Xe, and N_2_) cause inhibition of protein activity and that this activity could be recovered after degassing.

## Results

**Figure 1:**
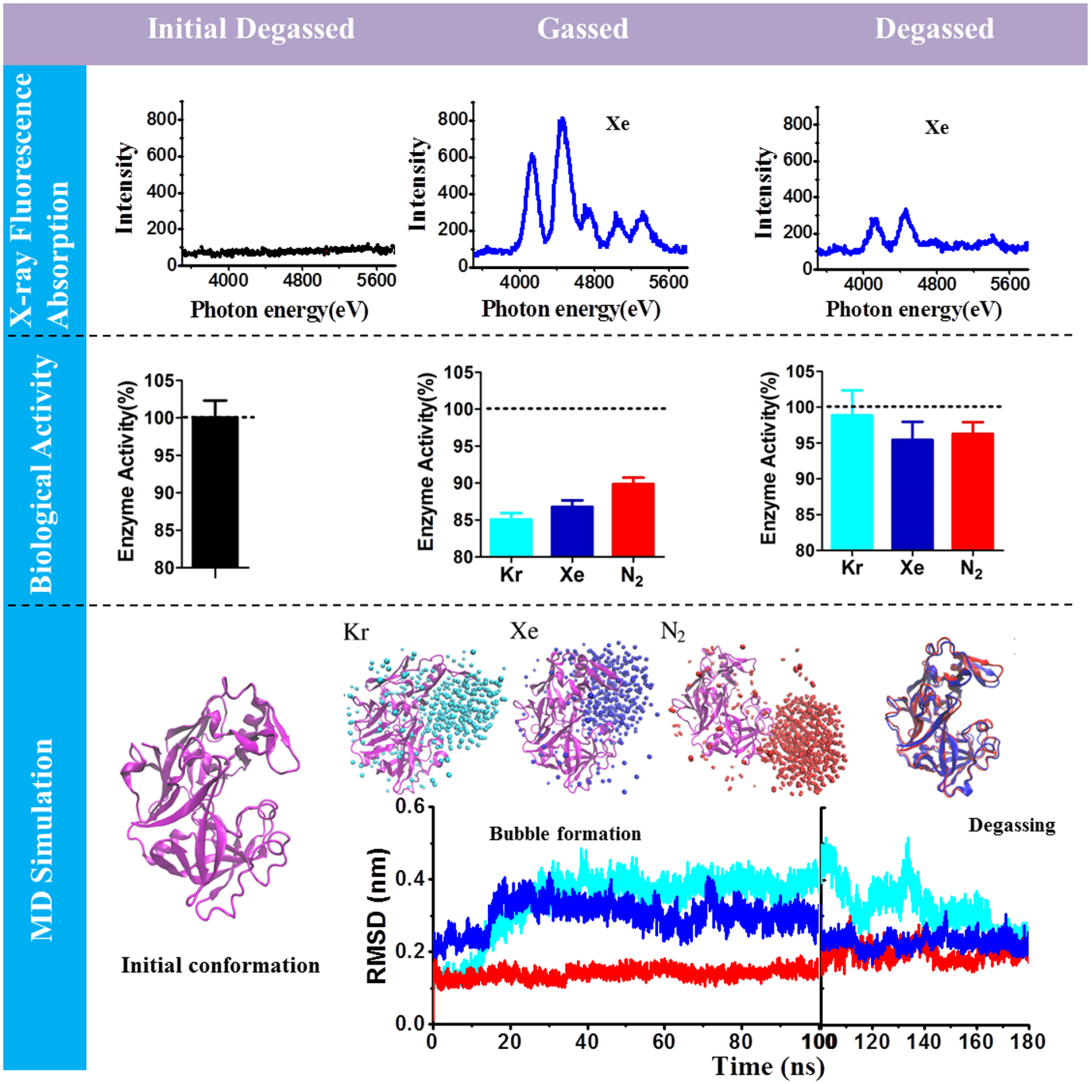
Outline and representative results in this study. The upper row shows micro X-ray fluorescence measurements in the pepsin system during the saturated gassed and degassed states, using Xe as an example. From left to right, the black and blue curves refer to the absorption of the pepsin system (0.5 mg/ml) without Xe and after gassing with Xe, respectively. The right curve shows the absorption of Xe in the pepsin solution after degassing for about 2 hours. The middle row shows the activity of pepsin measured in the saturated gas and degassed states together with the control system. The bottom row displays the MD simulation results of the backbone root-mean-square deviation (RMSD) and the conformations of pepsin with Kr, Xe, and N_2_ aggregations/bubbles and after degassing. In all results the black colour shows the data of the initial degassed system while the light blue, blue, and red colours represent the data of Kr, Xe, and N_2_.

Figure 1 provides the main outline and some results of the study.

### Gas molecule concentration in pepsin solution detected using micro X-ray fluorescence absorption spectra

The concentration of Xe and Kr dissolved in a pepsin solution was studied by measuring the micro X-ray fluorescence absorption near the Xe L- edge and Kr K-edge of pepsin solutions with two concentrations (0.1 mg/ml and 0.5 mg/ml) after gassing and degassing of Kr and Xe. The degassed water and HCl solution without pepsin were used as controls.

**Figure 2:**
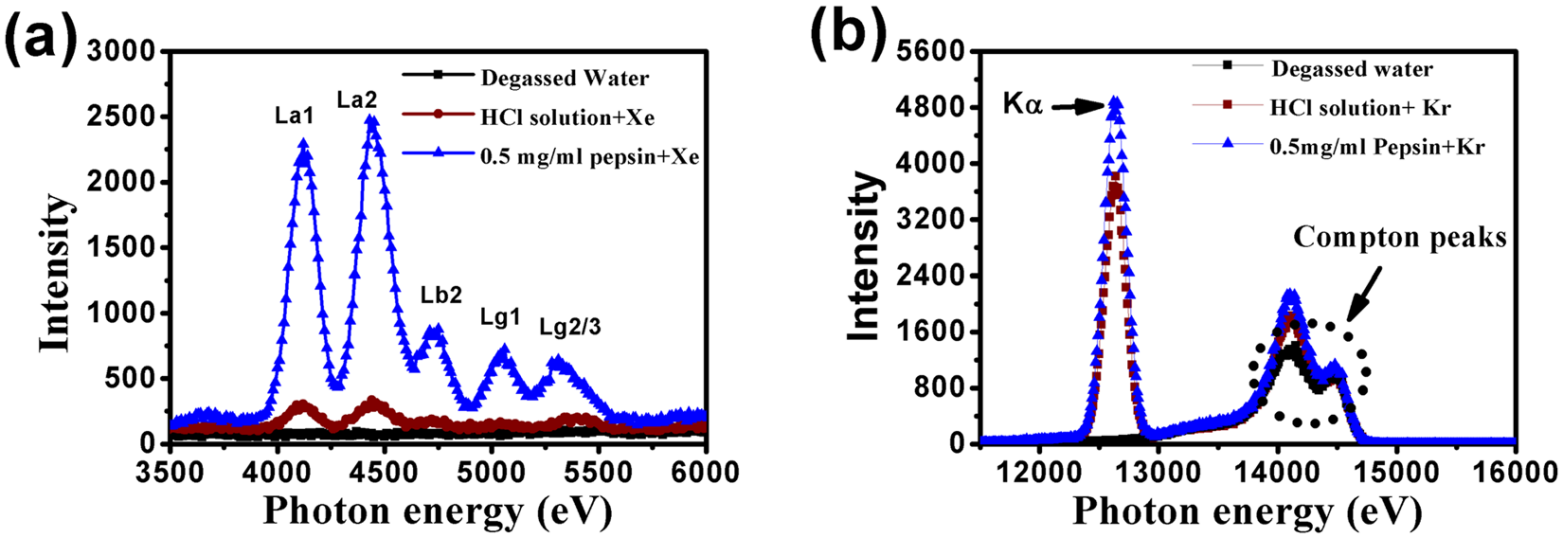
Micro X-ray fluorescence absorption near (**a**) Xe L-edge and (**b**) Kr K-edge in a pepsin solution, HCl solution without pepsin, and degassed water.

Figure 2 shows the micro X-ray fluorescence absorption of Xe and Kr in 0.5 mg/ml pepsin, an HCl solution without pepsin, and degassed water. Typical Xe *L* X-ray spectra are measured to resolve the individual *Lα* (or *Lβ*) X-ray transitions^24, 25^ as showed in Fig. 2a. The fluorescence absorption of Xe with pepsin is about 10 times stronger than that in the HCl solution without pepsin, suggesting a much higher concentration of Xe dissolved in the pepsin solution. For the Kr system, there is also a higher concentration of Kr in the pepsin solution than in the HCl-only solution (Fig. 2b).

**Figure 3:**
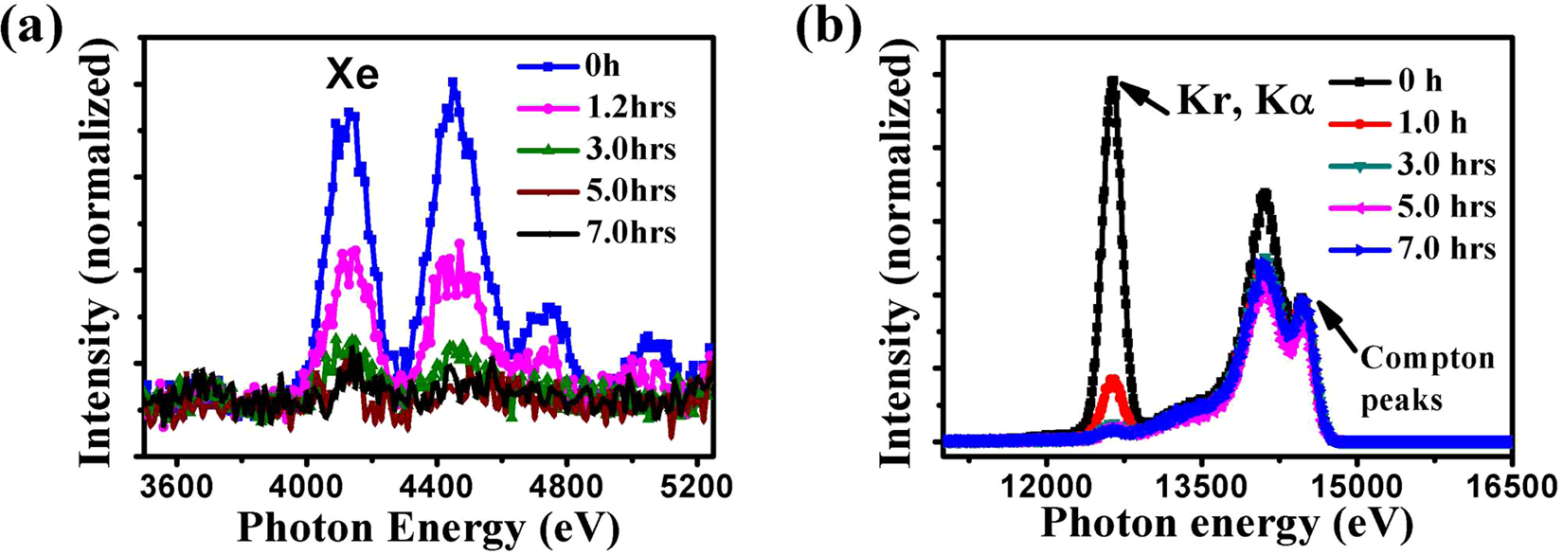
Micro X-ray fluorescence absorption of (**a**) Xe and (**b**) Kr with degassing time in a solution of 0.1 mg/ml pepsin.

The variation of Xe and Kr fluorescence absorption with time also measured at a concentration of 0.1 mg/ml pepsin solution (Fig. 3). The results indicate that the intensity of Xe in this system decreases very quickly at the beginning (before 3.0 hours) and then reaches a relatively low level (Fig. 3a). Similar results at the top right of Fig. 1, showing that the absorption intensity of Xe decreases in a 0.5 mg/ml pepsin system after degassing for about 2 hours, indicate that the observations may be universal for a range of pepsin concentrations. The absorption intensity of Kr K-edge (Kα) is strong at the beginning, but decreases quickly with degassing time, such as 1 hour (Fig. 3b). Compared to a Xe system, Kr may have a faster release time. After 1 hour the intensity decreased by 50% for the pepsin-containing Xe system but 82% for the pepsin-containing Kr system. This fast release of Kr is consistent with the experimental observation that the activity of pepsin in the Kr degassed state recovers quickly compared to the activity in the Xe degassed state (see Fig. 1).

In order to explore the distribution of inert gases in the pepsin solution, the solutions were mapped by X-ray fluorescence, using Xe as an example, (Supplementary Fig. 1). The result shows a non-uniform distribution of Xe in the pepsin solution, suggesting some gas aggregations in the local area although the spatial resolution of mapping is only several microns.

### Reduction of biological activity of pepsin due to gassing and recovery under degassing of biologically inert gases

The biological activity of pepsin under gassing and degassing was investigated to examine the influence of a high concentration of Xe or Kr on the activity in a pepsin solution (Fig. 2), moreover whether this activity can be recovered after degassing. The middle row of Fig. 1 shows that the relative enzymatic pepsin activity decreases to about 85.1 ± 0.9%, 86.8 ± 0.9%, and 89.9 ± 0.9% with Kr, Xe, and N_2_ saturated gas, respectively. Activity recovery experiments were performed to further test if gassing causes the decrease in enzyme activity. The samples with initial saturated gases are gently oscillated for 2 hours before adding a hemoglobin substrate for the follow-up activity measurement. As shown in Fig. 1, the pepsin activities increase to 98.9 ± 3.5%, 95.4 ± 2.5%, and 96.3 ± 1.6%, revealing a near complete recovery from the inhibition. Over all, the relative activity of pepsin could be reversed by gassing and degassing, suggesting that biologically inert gases can significantly manipulate the protein activity. It is worth noting that the trend in the reduction of pepsin activity by the three gases in the saturated gas case has the ordering Kr > Xe > N_2_, and the recovery in the Kr and Xe systems shares the same order.

### Nanoparticle tracking analysis

**Figure 4:**
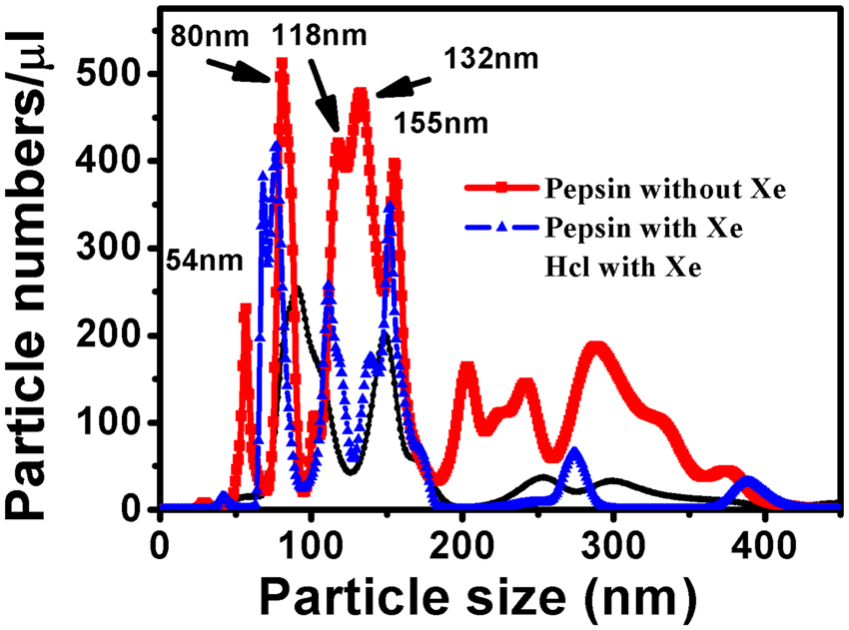
The distribution of “particles” measured in a pepsin solution with and without Xe as well as Xe saturated HCl solution. The distribution of nanoparticles differs from the system without Xe. From the curves, the number of “particles”, especially nanometre-scale particles, increases after injecting Xe in the pepsin solution.

The Xe system is further examined to determine whether nanoscale gas bubbles form in the pepsin solution after gassing. The distribution of “nanoparticles” was followed by tracking analysis based on dynamic light scattering^26^. Figure 4 shows particle distribution in a 0.1 mg/ml pepsin solution after gassing for about 2 hours in a Xe atmosphere, as well as in the pepsin solution before Xe gassing. The system of Xe saturated solution without pepsin was also presented to compare with the system with pepsin. It was found that the size distribution of “particles” measured in the pepsin solution with Xe is quite different from that in the systems without Xe. The number of “particles” increases after gassing with Xe, which is consistent with micro X-ray fluorescence absorption results. More importantly, “particles” with sizes 54 nm, 80 nm, 118 nm, 132 nm, and 155 nm dominate the distribution after gassing, and it could be presumed that a large number of nanobubbles form in the pepsin solution. The distribution of those peaks and number of “particle” are different from the system of Xe saturated solution without pepsin even they also can form nanobubbles in solution. The change in particle distribution over time was also measured at different times within a Xe atmosphere. This showed the number of “particles” smaller than 100 nm increased at times within a range of 70–210 min, also indicating the formation of numerous gas “nanoparticles” (Supplementary Fig. 2). In our experiment we could detect the “nanoparticles” of about 20 nm but much smaller ones could not observed due to the limit of the resolution of NanoSight.

### MD simulations of the dynamics of the interactions between biologically inert gas molecules and pepsin

MD simulations were performed to study the binding of an inert gas to pepsin and the structural changes of pepsin induced by the inert gases. To identify the minimum bubble formation concentration of Kr, Xe, and N_2_ in the simulation systems, several MD simulations were performed for each gas by increasing its concentration until a gas bubble was observed. As listed in Table 1 and shown in Fig. 1, when the numbers of Kr, Xe, and N_2_ reach 600, 600, and 750, respectively, an aggregation is formed around the pepsin. Both the Kr and Xe bubbles form at the active site of pepsin (see bottom row in Fig. 1). Consequently, the Kr and Xe bubbles invade the active site and hinder the access of the substrates. Moreover, in the case of Kr, the conformation of pepsin at the active site is greater than it is in the case of Xe, which can be clearly observed by the RMSD analysis (bottom row of Fig. 1). However, unlike Kr and Xe, the N_2_ bubble is formed at the surface region of pepsin, which may hinder the entrance of the cavity sterically. It is presumed that both Kr and Xe need smaller seeds to induce bubble aggregation within the active site, while the N_2_ aggregates around the hydrophobic active site are due to the need for a larger seed to make the bubble grow. The RMSD analysis also shows that the N_2_ bubble has much less influence on the conformation of pepsin at the active site. The extent of deformation at the active site affected by the gas bubble (Kr > Xe > N_2_), is in agreement with the activity assay mentioned above: Kr exhibits the largest activity inhibition, while N_2_ results in the smallest inhibition.

**Table 1:** MD System parameters (number of molecules).

MD simulation systems	Gas type	Gas number	Water	Pespin	Na/Cl
1	None	0	28473	1	54/58
2	Kr	600	27675	1	54/58
3	Xe	150	28265	1	54/58
4	Xe	300	28064	1	54/58
5	Xe	600	27665	1	54/58
6	N_2_	300	27884	1	54/58
7	N_2_	500	27459	1	54/58
8	N_2_	750	27061	1	54/58

**Figure 5:**
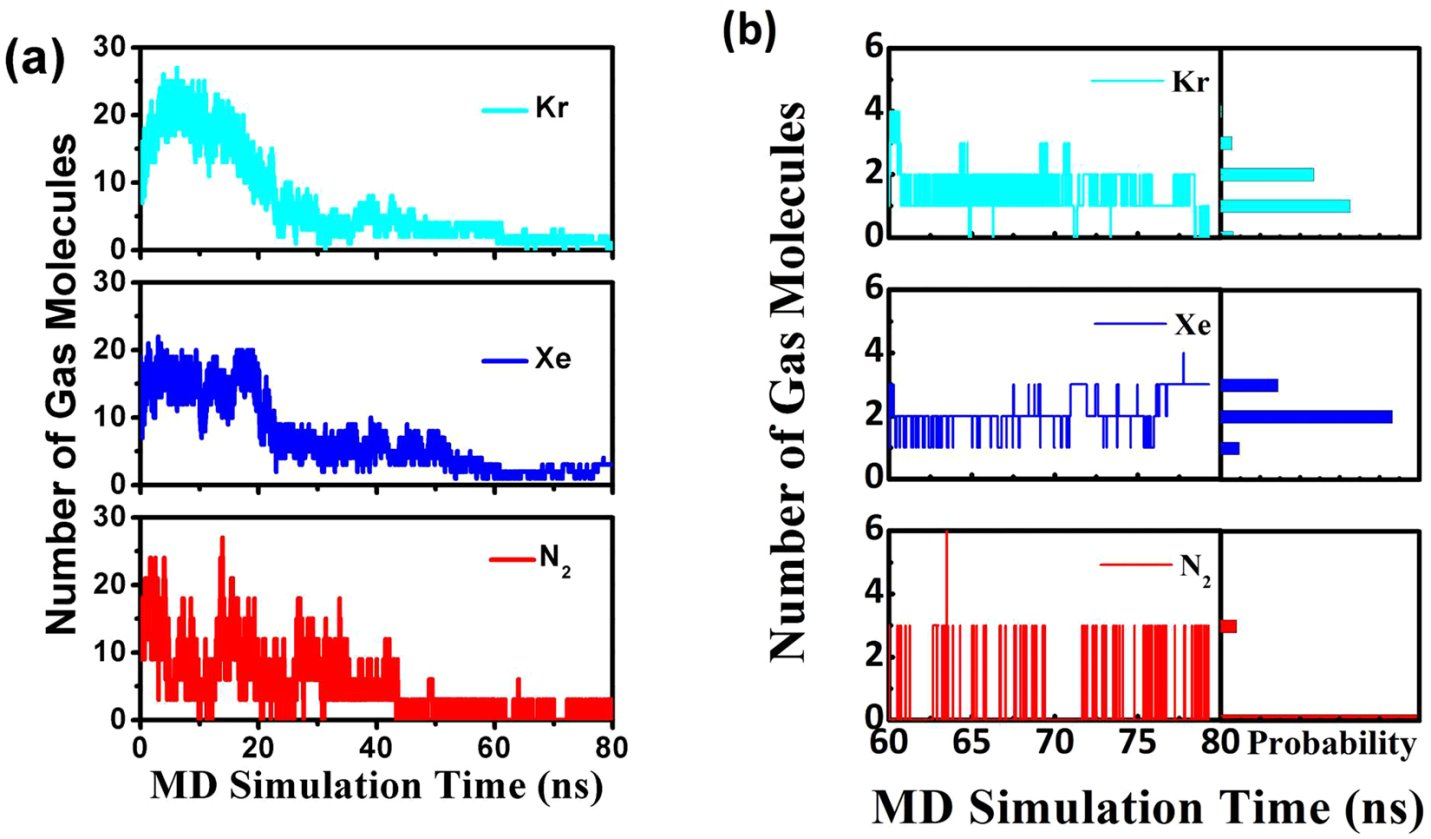
(**a**) MD simulations of the degassing process over 80 ns. (**b**) Enlargement showing the range from 60 ns to 80 ns.

MD simulations were also conducted to mimic the degassing process using the water replacement protocol mentioned in the Method section. As shown in Fig. 5a, at the beginning of our degassing MD simulations, both Kr and Xe aggregate heavily around the active site-there are more than 20 gas molecules within 10 Å of the active site. Interestingly, although the N_2_ bubble does not form at the active site, a considerable but fluctuating number of N_2_ molecules is observed near the active cavity, which further explains the activity inhibition of pepsin by N_2_ gas. During the MD simulations of degassing, the Kr and Xe numbers around the active site decline rapidly in the first 25 ns and become very small after 60 ns. Figure 5b shows that, most of the time from 60 ns to 80 ns, Kr, Xe, and N_2_ exhibit gas numbers of 1, 2, and 0 around the active site. This is consistent with both experiments on pepsin activity recovery and the decrease in the gas molecule concentration in the pepsin solution, detected using micro X-ray fluorescence absorption spectra after degassing. After 80 ns of degassing, all conformational changes in pepsin influenced by inert gases have been recovered (bottom row of Fig. 1). The 4-strand anti-parallel β sheets at the active site move back to their native conformation, indicating restored activity. We note that Xe molecules are frequently observed in the protein crystals, which may correspond to the degassing phases here and should not be used against the formation of Xe bubbles in biological processes.

## Discussion

Combining from X-ray fluorescene absorptions and protein activity measurements revealed several physical interactions between the inert gases and pepsin. It was demonstrated that Kr releases faster than Xe while degassing the pepsin solution. This may be explained by the free energy of binding of bubbles with the inhibitor pepstain as showed in Table S1. The Xe bubble demonstrates the highest binding free energy of −16.08 kcal/mol and the Kr bubble shows a comparable inhibitory property with the inhibitor pepstain. The N_2_ bubble exhibits the smallest binding affinity among three gas bubbles. Another point of interest is that different gases exhibit different inhibition for pepsin activity. That is to say, the trend in the reduction of pepsin activity by the three gases in the saturated gas case has the ordering Kr > Xe > N_2_, and the recovery in the Kr and Xe systems shares the same order. This might be understood from the different binding sites of gas with pepsin molecules as above MD results. N_2_ molecules do not aggregate near the hydrophobic active cavity of pepsin which leads to less inhibition on pepsin activity. Our RMSF (root mean square fluctuations) data also showed in Fig. 4 further explains the inhibitory mechanisms of pepsin in the presence of different inert gases.

## Conclusions

X-ray fluorescence absorptions of Xe and Kr are significantly stronger in Xe- or Kr-injected pepsin solutions compared to the case without pepsin, suggesting a higher concentration of Xe or Kr dissolved into the pepsin than in a normal protein-free solution. Further pepsin activity experiments show that the biologically inert gases Kr, Xe, and N_2_ have an evident inhibition on the biological activity of pepsin, and this effect could be almost fully recovered after the release of these gases. Interestingly, the extent of the inhibition and recovery of pepsin activity differs according to which gas. X-ray fluorescence is also supported by nanoparticle size measurements which indicate a higher number of “nanoparticles” in gas-containing pepsin solution. MD simulations further show that Kr, Xe, and N_2_ gas molecules aggregate near the active residues as bubble shapes, suggesting that the reduced activity of the pepsin is due to the existence of gas molecule aggregations. Further simulation results indicate that the degassing switches the aggregation state into a dispersed state, implying the reversibility of the gas function. More importantly, both experiments and MD simulations share that the degrees of activity reduction in the saturated gas state show the ordering of Kr > Xe > N_2_, in agreement with the proposed molecular mechanism. Combining these observations, the aggregated gas molecules on the active residues result in a functional reduction in the pepsin. This work not only provides fundamental insights into the functional roles of inert gases in biological systems, but also increases our understanding of the mechanism of anaesthetics due to biologically inert gases.

## Methods

### Micro X-ray fluorescence experiments

The x-ray absorption of Xe and Kr in pepsin solution was measured by micro X-ray fluorescence mapping and absorption at the beamline 15 U in Shanghai Synchrotron Radiation Facility (SSRF). The photon energy was set to be 10.0 keV for Xe and 14.5 keV for Kr measurements, respectively. The flux was about 10^11^ photons/s at the sample. A focused beam with spot size of about 2.2 × 2.5 µm^2^ was used to excite the sample. The elemental distribution was acquired by raster-scanning the sample. The step size was set to be 2.0 µm and the collecting time for each step was 20 ms. Firstly, the water was degassed for about 1.5 hours for removing dissolved gas in water. Then the concentration of pepsin in HCl buffer solution (0.065 mol/L) was prepared to 0.1 mg/ml as activity measurements. Another 0.5 mg/ml pepsin solution was chosen for comparison. Those pepsin solutions were kept in Xe atmosphere for 30 mins in a sealed chamber at about 10 atms. Then releasing pressure to one atmosphere and several microliter pepsin solution was quickly taken and filled in liquid cell (with thickness of about 0.5 mm). The measurements of micro X-ray fluorescence absorption were performed at one atmosphere and room temperature. Degassed water and HCl solution without pepsin were also measured for comparison. Those experiments were repeated three times for each sample.

### Pepsin activity measurement

The enzyme activity was determined by the Anson method with some modifications^27^. Briefly, Pepsin (0.1 mg/ml, purchased from Sigma-Alrich) in 0.065 mol/L HCl buffer was gassed with various gases (Kr, Xe and N_2_ purchased from Maxtor’s special gas corporation, LTD., Shanghai) at 37 °C under 1.3 MPa pressure for 2.0 hours, and then 5.0 mL of 1% bovine hemoglobin (purchased from National Institutes for Food and Drug Control) solution was added in. After 10 mins, 5.0 ml of 5% trichloroacetic acid was added to terminate the above reaction. The mixture was put there for 15 mins statically and then centrifuged at 9000 rpm for 15 mins. The obtained supernatant was measured via OD 275 nm using a UV-Vis spectrophotometer (HTACHI corporation, U-3010 spectrophotometer). The relative activities of pepsin in the presence of gases were obtained by the following equation: Relative activity = OD275 (pepsin-gas)/OD275 (pepsin).

### Nanoparticle tracking analysis

A NanoSight (NS300, Malvern) instrument for nanoparticle tracking analysis using a blue laser light source (70 mW, λ = 405 nm) at room temperature was used to measure the sizes and numbers of “nanoparticles” in pepsin solution. The Nanosight determines the size of individual particles from their diffusion under Brownian motion using nanoparticle tracking. The detected range of nanoparticles is 10 nm–2000 nm. The concentration with sizes is directly determined by counting the number of particles observed in a known volume. The tracking of numerous particles enables the size distribution of particles to be determined. Gassing experiments were performed in a sealed chamber and solution was kept in Xe atmosphere for different times. Several microliters pepsin solution was quickly taken and filled slowly into the pre-cleaned liquid cell with syringe, then five movies at five injections, each for 60 s long, were captured at 25 frames/s. Equipped with the analysis software, NTA 3.2 Dev Build 3.2.16, the camera level was set to15–20 for each time, the gain to 10, and analysis was conducted using a solution viscosity of water (1.00 cP).

### MD simulations

The initial conformation of pepsin was taken from the crystal structure of human pepsin (PDBid: 1psn). MD simulations were conducted by employing the GROMACS 4.6.5 package and OPLS all-atom force field. Several MD systems, listed in Table 1, were prepared for investigating the bubble formation in the presence of Kr, Xe and N_2_, respectively. First, the protein was centered into a periodic cubic box with the size of 9.67 × 9.67 × 9.67 nm^3^ and the gas molecules were randomly dispersed into the box around the protein. The force field parameters of Kr, Xe and N_2_ used in this work are listed in Table 2. Note that a three-charge-site N_2_ model was adopted here and a massless dump atom set between the N–N atoms to account for the quadrupole moment. Then, the systems were dissolved with tip3p water molecules. All the Asp and Glu residues of pepsin were protonated as well as the His residues which were protonated on both NE2 and ND1 atoms. 0.1 M NaCl was used to balance the charge of the whole system. After a short time of energy minimization, the systems were equilibrated in *NPT* ensemble for 1 ns. Then, the production runs were performed in *NVT* ensemble for 100 ns at 300 K. The bond lengths attached with hydrogen atom were constrained by using the LINCS algorithm. A time step of 2 fs was used in all simulations. The particle-mesh Ewald method was used for the calculation of long-range interactions with a cutoff of 1.2 nm.

**Table 2:** Force field parameters for gas molecules Kr, Xe and N_2_, respectively.

	ε/k_B_ (K)	σ(Å)	q (e)	Bond length(Å)
Kr^28^	169.0	3.675	0	
Xe^29^	214.7	3.975	0	
N^30^	36.4	3.318	−0.4048	
Center-Of-Mass	0	0	0.8096	
N-N				1.098

For the MD simulations of the degassing process, we used the water replacement protocol (see Figure S3). First, we extended the original simulation box (Box_A) along x-axis to double the system volume using the last frame from the 100 ns production run. Then we filled the empty area with TIP3P water molecules. After performing energy minimization and a short equilibration, a 10 ns NVT MD simulation was performed to let the gas molecules diffuse in the dual-sized simulation box (Box_AB). Finally, we purged the water and gas molecules in Box_B and refilled the system with new TIP3 water molecules. The process was repeated several times until the number of the gas molecules around pepsin reaches an equilibrium.

## Electronic supplementary material

Supplementary materials

## References

[1] Dingley J, Ivanova-Stoilova TM, Grundler S, Wall T (1999). Xenon: recent developments. Anaesthesia.

[2] Winkler DA, Thornton A, Farjot G, Katz I (2016). The diverse biological properties of the chemically inert noble gases. Pharmacology & Therapeutics.

[3] Ewing GJ, Maestas S (1970). The thermodynamics of absorption of xenon by myoglobin. J. Phys. Chem..

[4] Schiltz M, Fourme R, Broutin I, Prange T (1995). The catalytic site of serine proteinases as a specific binding cavity for xenon. Structure.

[5] Morrison CN, Hoy JA, Zhang L, Einsle O, Rees DC (2015). Substrate Pathways in the Nitrogenase MoFe Protein by Experimental Identification of Small Molecule Binding Sites. Biochemistry.

[6] Schoenbo B (1965). Binding of xenon to horse haemoglobin. Nature.

[7] Malashkevich VN, Kammerer RA, Efimov VP, Schulthess T, Engel J (1996). The crystal structure of a five-stranded coiled coil in comp: A prototype ion channel?. Science.

[8] Prangé T (1998). Exploring hydrophobic sites in proteins with xenon and krypton. Proteins: Struct. Funct. Genet..

[9] Hayakawa N (2008). Effect of Xenon Binding to a Hydrophobic Cavity on the Proton Pumping Cycle in Bacteriorhodopsin. J. Mol. Biol..

[10] Luna VM, Chen Y, Fee JA, Stout CD (2008). Crystallographic Studies of Xe and Kr Binding within the Large Internal Cavity of Cytochrome ba3 from Thermus thermophilus: Structural Analysis and Role of Oxygen Transport Channels in the Heme-Cu Oxidases. Biochemistry.

[11] Tetreau C, Blouquit Y, Novikov E, Quiniou E, Lavalette D (2004). Competition with xenon elicits ligand migration and escape pathways in myoglobin. Biophys. J..

[12] Rubin SM, Spence MM, Goodson BM, Wemmer DE, Pines A (2000). Evidence of nonspecific surface interactions between laser-polarized xenon and myoglobin in solution. Proc. Natl. Acad. Sci. USA.

[13] Rubin SM, Spence MM, Pines A, Wemmer DE (2001). Characterization of the effects of nonspecific xenon-protein interactions on ^129^ Xe chemical shifts in aqueous solution: further development of xenon as a biomolecular probe. Journal of Magnetic Resonance.

[14] Franks NP, Dickinson R, de Sousa SLM, Hall AC, Lieb WR (1998). How does xenon produce anaesthesia?. Nature.

[15] Turin, L., Skoulakis, E. M. C. & Horsfield, Andrew P. Electron spin changes during general anesthesia in Drosophila. *Proc. Natl*. *Acad. Sci. USA* E3524–E3533 (2014). 10.1073/pnas.1404387111 PMC4151765 25114249

[16] Fenn WO (1965). Inert gas narcosis. Ann. NY Acad. Sci..

[17] Thomas JR, Burch LS (1975). Inert gas narcosis: Avoidance behavior in rats breathing elevated pressures of nitrogen and helium. Physiological Pgychology.

[18] Wang Y, Chen L, Wang X, Dai C, Chen J (2015). Effects on lipid bilayer and nitrogen distribution induced by lateral pressure. J Mol Model.

[19] Liu LT, Xu Y, Tang P (2010). Mechanistic Insights into Xenon Inhibition of NMDA Receptors from MD Simulations. J. Phys. Chem. B.

[20] Cao Q (2015). Interaction of Aromatic Compounds with Xenon: Spectroscopic and Computational Characterization for the Cases of p-Cresol and Toluene. J. Phys. Chem. A.

[21] Mons M, Calvé JL, Piuzzi F, Dimicoli I (1990). Resonant two-photon ionization spectra of the external vibrational modes of thechlorobenzene-, phenol-, and toluene-rare gas (Ne, Ar, Kr, Xe) van der Waals complexes. J. Chem. Phys..

[22] Ullrich S, Tarczay G, Müller-Dethlefs K (2002). Resonance-Enhanced Multiphoton Ionization and Zero Kinetic Energy Photoelectron Study of the Phenol· Kr and Phenol· Xe van der Waals Complexes. J. Phys. Chem. A.

[23] Bardhan, K. D., Strugala, V. & Dettmar, P. W. Reflux revisited: advancing the role of pepsin. *International Journal of Otolaryngology* 646–901 (2012). 10.1155/2012/646901 PMC3216344 22242022

[24] Romano C, Dingwell DB, Lechtenberg F (1998). Synchrotron x-ray fluorescence analysis of gas bubbles in glass. Phys. Chem. Glasses.

[25] Dias THVT (1997). Full-energy absorption of x-ray energies near the Xe L- and K-photoionization thresholds in xenon gas detectors: Simulation and experimental results. J. Appl. Phys..

[26] Zhu J (2016). Cleaning with Bulk Nanobubbles. Langmuir.

[27] Anson DL (1938). The estimation of pepsin, trypsin, papain, and cathepsin with haemoglobin. J. Gen. Physiol..

[28] Paschek D (2004). Temperature dependence of the hydrophobic hydration and interaction of simple solutes: An examination of five popular water models. J. Chem. Phys..

[29] Cohen J, Arkhipov A, Braun R, Schulten K (2006). Imaging the migration pathways for O, CO, NO, and Xe inside myoglobin. Biophysical Journal.

[30] Murthy CS, Singer K, Klein ML, McDonald IR (1980). Pairwise additive effective potentials for nitrogen. Molecular Physics: An International Journal at the Interface Between Chemistry and Physics.

