# Supplementary material for: Inert Gas Deactivates Protein Activity by Aggregation
Source: Sci Rep. 2017 Aug 31;7:10176. doi: 10.1038/s41598-017-10678-3 (PMC5579012; doi:10.1038/s41598-017-10678-3)
Supplement: Supplementary file 1 — Supplementary materials [file 41598_2017_10678_MOESM1_ESM.doc]

**Supplementary Information**

**Inert Gas Deactivates Protein Activity by Aggregation**

Lijuan Zhang^a,b^, Yuebin Zhang^c^, Jie Cheng^a,e,f^, Lei Wang^b,g^, Xingya Wang^b,f^, Meng Zhang^a,d^, Yi Gao^a,d^, Jun Hu^a,e^, Xuehua Zhang^h^, Junhong Lü^a,e*^, Guohui Li^c*^, Renzhong Tai^a,b^, Haiping Fang^a,d*^

^a^ Key Laboratory of Interfacial Physics and Technology, Chinese Academy of Sciences, Shanghai 201800, China

^b^Shanghai Synchrotron Radiation Facility, Shanghai Institute of Applied Physics, Chinese Academy of Sciences, Shanghai 201204, China

^c^State Key Laboratory of Molecular Reaction Dynamics, Dalian Institute of Chemical physics, Chinese Academy of Sciences, Dalian 116023, China

^d^Division of Interfacial Water, Shanghai Institute of Applied Physics, Chinese Academy of Sciences, Shanghai 201800, China
^e^Division of Physical Biology, Shanghai Institute of Applied Physics, Chinese Academy of Sciences, Shanghai 201800, China

^f^University of the Chinese Academy of Sciences, Beijing 100049, China

^g^Institute of Mathematics and Physics, Central South University of Forestry and Technology, Changsha 410004, China

^h^School of Civil, Environmental and Chemical Engineering, RMIT University, Melbourne, VIC 3001, Australia


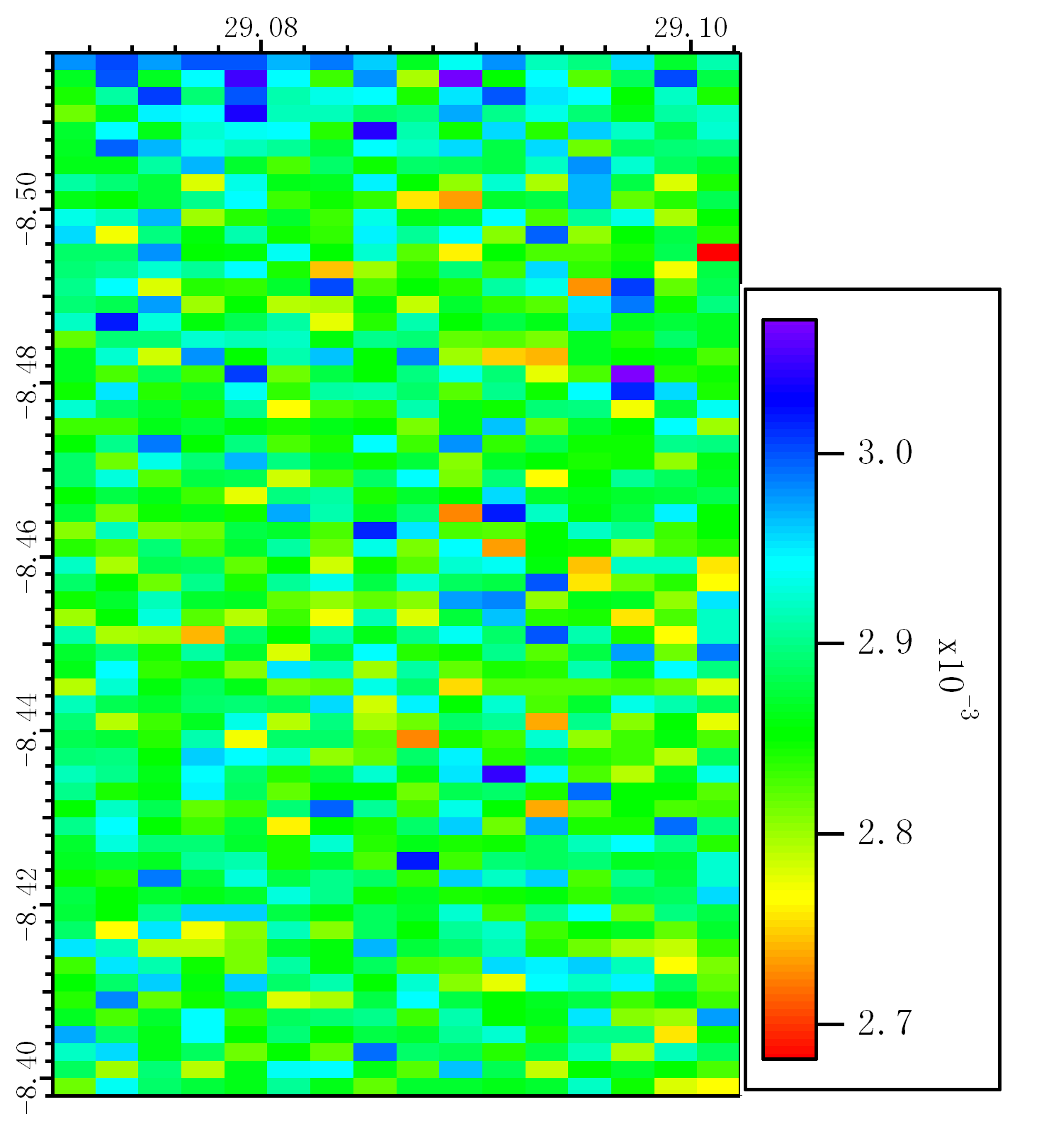


Figs. 1 Micro X-ray fluorescence mapping of Xe in 0.5mg/ml pepsin solution. The result shows that the distribution of Xe in pepsin solution is not uniform which might be some tiny bubbles or gas aggregations exist in the system. Scan size: 30μm×120μm.

We also perform the fluorescence mapping of Xe in different pepsin solution with different concentration to know the 2D distribution of Xe. Figure s1 presents the typical example. It is found that the distribution of Xe is not uniform even though this mapping can not distinguish single bubbles. But it indicated that many tiny bubbles might distribute different position in pepsin solution, which might cause the high concentration Xe as showed in Micro X-ray fluorescence absorption.

Figs.2 “Particle” numbers relative to their size distributions in pepsin solution with different keeping time in one Xe atmosphere. The “particles” with sizes below 100nm would increase while the keeping time is in the range of 70-210 mins. This might be caused by the formation of gas “nanoparticles” (nanobubbles).


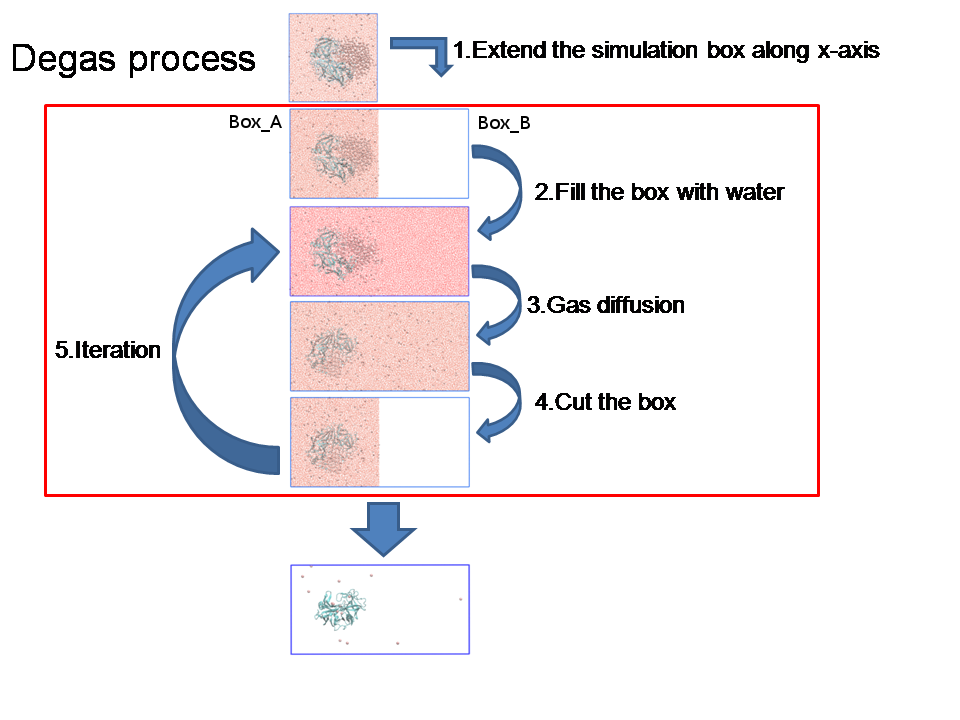


Figs.3 Water replacement protocol for simulations of the degassing process

1. The original simulation box(Box_A) was extended along x-axis to double the system volume using the last frame from the 100ns production run;

2. The empty box (Box_B) was filled with TIP3 water molecules;

3. After performing energy minimization and a short equilibration, 10ns NVT MD simulation was conducted to let the gas molecules diffuse in the dual-sized simulation box (Box_AB).

4. The water and gas molecules in Box_B were purged and the system was refilled with new TIP3 waters.

5. The process was repeated several times until the numbers of the gas molecules around pepsin reaches equilibrium.


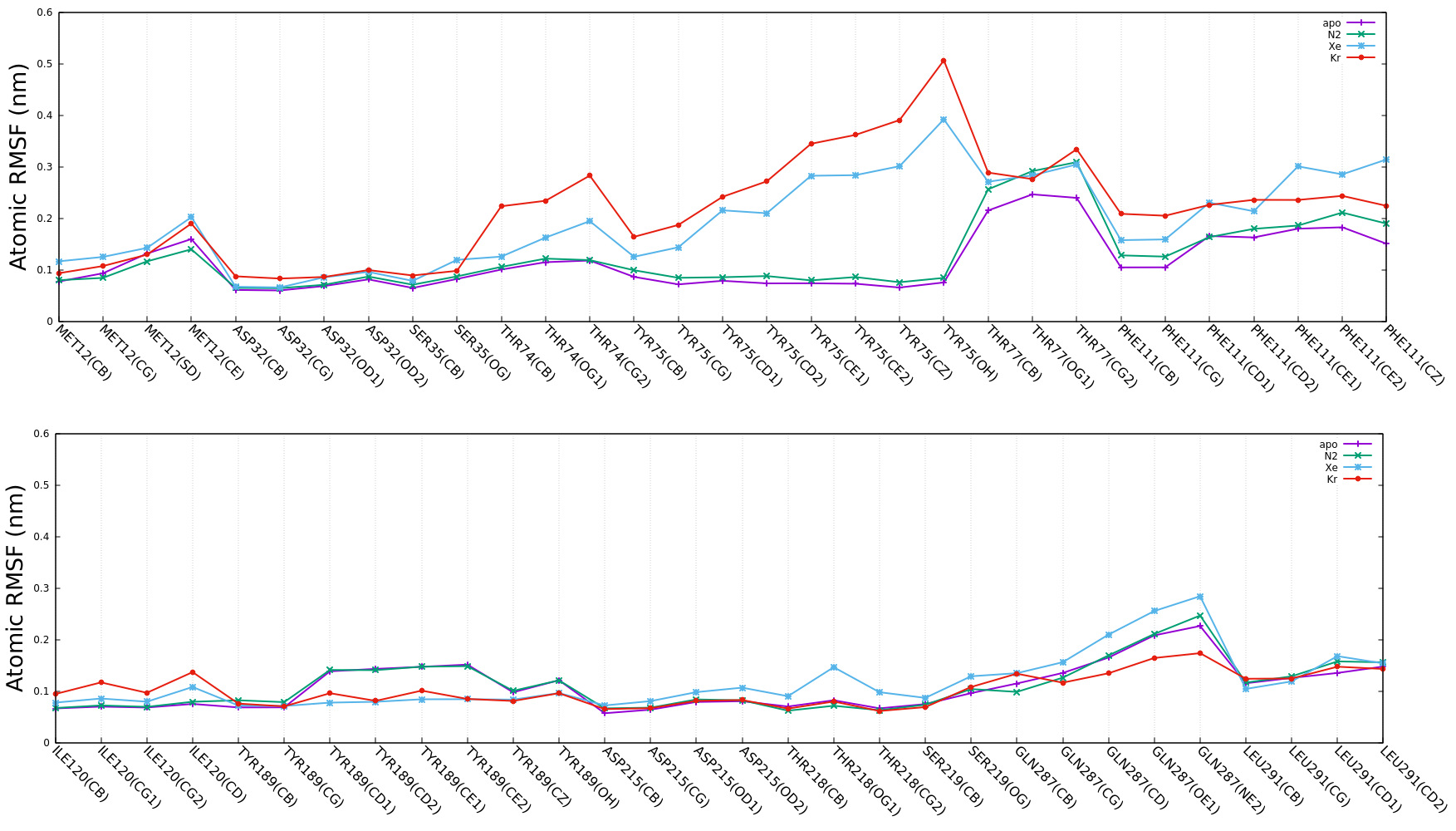


Figs.4 Atomic RMSFs (root mean square fluctuations) of side chains of the residues lining the active site trench of pepsin in the presence of Kr (red line), Xe (blue line), N_2_ (green line) and in the absence of gases (purple line). The active sites of pepsin were identified using the crystal structure of human pepsin with the inhibitor pepstain (PDBid:1PSO),where the residues within the distance of 5Å of the inhibitor pepstain (including the residues of M12 ,D32 ,G34 ,S35, T74, Y75, G76, T77, F111, I230, Y189, D215, G217, T218,S219, Q287 and L291) were selected as active sites and the atomic RMSFs of the side chains of those residues were calculated using 100ns MD trajectories.

According to Figs.4, we can observe that the highly conversed tyrosine residue located on the beta hairpin loop over the active side cleft (so-called “flap”) at position 75, especially for the oxygen atom of the hydroxyl group of Y75, is dramatically influenced in the presences of both Kr and Xe gases. This flap plays a significant role in pepsin catalysis, which opens to allow substrate access to the active site trench, closes on substrate binding and opens again to allow products to leave. Whereas in the presence of N_2_ gases, the flap is less affected compared with the RMSFs in the absence of gases because that the N_2_ bubble is not aggregated around the hydrophobic active cavity of pepsin. Our RMSF data further explains the inhibitory mechanisms of pepsin in the presence of inert gases.

Table S. 1 Estimated free energy of binding & inhibition constant

| Ligands | Free energies of binding | Inhibition Constants  T = 295.15 K |
| --- | --- | --- |
| pepstain | -12.80 kcal/mol | 414.80 pM |
| Xe bubble | -16.08 kca/mol | 1.63 pM |
| Kr bubble | -11.78 kca/mol | 2.33 nM |
| N_2_ bubble | -8.51 kcal/mol | 557.24 nM |

In Table S.1, the free energy of binding and the inhibition constants were estimated using AutoDock Vina, which have been successfully used to fast estimate the binding affinity of a ligand to a protein for a given conformation.

In this study, we treated the nanobubbles formed around the surface region of pepsin as a ligand of Lennard-Jones particles.

The crystal structure of human pepsin with the inhibitor pepstain (PDBid: 1PSO) was employed to calculate the binding affinity of pepstain. For the binding affinity calculations of gases molecules, the last snapshots of our 100ns MD simulation trajectories were used. For bubble identifications, the coordination number of each gas molecule was counted using a distance cutoff value of 7Å and the gases with a coordination number larger than 8 were grouped as the bubble. Same force filed parameters of inert gases listed in table 2 were used for estimating free energies of binding.

As shown in Table S. 1, the estimated binding affinity of pepsin to the ligand pepstain is 414.80 pM (picomolar), which agrees well with the experimental observation that the ligand pepstain exhibits a strong inhibitory property to pepsin at picomolar concentrations. The Xe bubble demonstrates the highest binding free energy of -16.08 kcal/mol and the Kr bubble shows a comparable inhibitory property with the inhibitor pepstain. The N_2_ bubble exhibits the smallest binding affinity among three gas bubbles.
